# Xylem Vessel Diameter Affects the Compartmentalization of the Vascular Pathogen *Phaeomoniella chlamydospora* in Grapevine

**DOI:** 10.3389/fpls.2017.01442

**Published:** 2017-08-21

**Authors:** Jérôme Pouzoulet, Elia Scudiero, Marco Schiavon, Philippe E. Rolshausen

**Affiliations:** ^1^Department of Botany and Plant Sciences, University of California, Riverside, Riverside CA, United States; ^2^United States Salinity Laboratory, United States Department of Agriculture–Agricultural Research Service, Riverside CA, United States

**Keywords:** compartmentalization, *Vitis vinifera* L. (grapevine), *Phaeomoniella chlamydospora*, plant vascular system, tylosis, wilt disease, xylem

## Abstract

Fungal wilt diseases are a threat to global food safety. Previous studies in perennial crops showed that xylem vessel diameter affects disease susceptibility. We tested the hypothesis that xylem vessel diameter impacts occlusion processes and pathogen compartmentalization in *Vitis vinifera* L. We studied the interaction between four grape commercial cultivars with the vascular wilt pathogen *Phaeomoniella chlamydospora*. We used qPCR and wood necrotic lesion length to measure fungal colonization coupled with histological studies to assess differences in xylem morphology, pathogen compartmentalization, and fungal colonization strategy. We provided evidence that grape cultivar with wide xylem vessel diameter showed increased susceptibility to *P. chlamydospora*. The host response to pathogen included vessel occlusion with tyloses and gels, deposition of non-structural phenolic compounds and suberin in vessel walls and depletion of starch in parenchyma cells. Pathogen compartmentalization was less efficient in wide xylem vessels than in narrow diameter vessels. Large vessels displayed higher number of tyloses and gel pockets, which provided substrate for *P. chlamydospora* growth and routes to escape occluded vessels. We discuss in which capacity xylem vessel diameter is a key determinant of the compartmentalization process and in turn grape cultivar resistance to disease caused by *P. chlamydospora*.

## Introduction

Wilt-diseases caused by fungal vascular pathogens such as *Fusarium* or *Verticillium* impact the productivity of annual and perennial crops worldwide and represent a serious threat to global food safety (Mace et al., 1981; Yadeta and Thomma, 2013). In woody angiosperm, signs of infection by wilt pathogens may range from the acute form, characterized by a sudden collapse of the plant during a single growing season, to the mild or chronicle form with symptoms that are progressive or intermittent from year to year (Talboys, 1968). Other characteristics of wilt causing fungi include a spatial limitation to the lumen of plant xylem vessels with both passive and active colonization of the host by mean of spores transported in the xylem sap flow and hyphal growth, respectively (Mace et al., 1981; Yadeta and Thomma, 2013). As these pathogens colonize the plant vascular system, the water transport function becomes increasingly compromised due to the occlusion of xylem vessels in response to infection. The translocation of fungal phytotoxins in the plant evapo-transpiration stream can also participate to the host decline and appearance of wilt symptoms (Tjamos and Beckman, 1988). Most fungal wilt pathogens are soil-borne or vectored by insects (Mace et al., 1981; Soulioti et al., 2015). The control of wilt diseases on perennial crops such as Olive tree (*Olea europaea*) consists in an integrated management strategy that combine the use of preventive, pre-planting and post-planting measures to minimize pathogen dispersion and risk of infection of new plant (Jimenez-Diaz et al., 2012; Yadeta and Thomma, 2013). Planting of cultivars resistant to wilt diseases remains one of the most durable and economically efficient control measure (Yadeta and Thomma, 2013), hence highlighting the necessity to better understand the plant defense mechanism and identify resistant genotypes within existing germplasms.

*Phaeomoniella chlamydospora* (W. Gams, P. Crous, M.J. Wingf., and L. Mugnai) (Crous and Gams, 2000) (*P. chlamydospora*) is a vascular wilt pathogen of cultivated grapevine, *Vitis vinifera* L., causing diseases known as esca and Petri disease (i.e., a young vine decline) (Larignon and Dubos, 1997; Mugnai et al., 1999; Feliciano et al., 2004; Gubler et al., 2004; Pouzoulet et al., 2014). *P. chlamydospora* is endemic to all viticulture areas worldwide and is responsible for significant economical losses to the grape industry (Bertsch et al., 2013; Bruez et al., 2013). Two forms of the esca disease can be observed in the field. The acute form, characterized by a sudden wilt of affected plants (a.k.a. apoplexy) that is favored during hot and dry summers, and the chronicle form characterized by the expression of progressive symptoms on leaves and berries, that can be intermittent from year to year (Mugnai et al., 1999; Guerin-Dubrana et al., 2012; Bertsch et al., 2013). *P. chlamydospora* possess all the traits of a vascular wilt pathogen including a systemic host colonization, spatial restriction to xylem vessels with limited ability to degrade structural plant cell wall polymers, and production of phytotoxins (Abou-Mansour et al., 2004; Bruno and Sparapano, 2006a,b,c; Santos et al., 2006; Valtaud et al., 2009; Fleurat-Lessard et al., 2010; Luini et al., 2010; Morales-Cruz et al., 2015). Several infection routes have been identified including infected nursery plant material (Whiteman et al., 2007), soilborne (Agusti-Brisach et al., 2013; Pouzoulet et al., 2013b) and airborne infections (Eskalen et al., 2007; Moyo et al., 2014). Field observations indicated that while no grape cultivar is immune to esca (i.e., absence of complete resistance), the incidence of the disease as measured by the expression of foliar symptoms vary across cultivars within a given geographic area (Bruez et al., 2013; Murolo and Romanazzi, 2014). Other studies reported that grape cultivars and rootstocks also differed in their degrees of susceptibility after experimental inoculation, as measured by differences in foliar symptom incidence, streaking in woody tissue, timing of bud breaking and shoot weight (Eskalen et al., 2001; Feliciano et al., 2004; Gramaje et al., 2010; Travadon et al., 2013).

Plant host genotypes may display resistance, tolerance or susceptibility to wilt pathogens (Beckman and Roberts, 1995; Fradin and Thomma, 2006). Resistance is characterized by the ability of the plant host to successfully compartmentalize the pathogen, whereas in tolerant and susceptible genotypes the plant host is not able to restrict pathogen movement often leading to systemic infection. However, tolerant plant genotype exhibits little disease symptoms despite pathogen colonization as oppose to susceptible plant genotypes suggesting that tolerant plants have the ability to counteract the effects of virulence factors produced by the wilt pathogens (Beckman and Roberts, 1995). The genetic basis of the resistance can be monogenic or polygenic (Perchepied et al., 2005; Fradin and Thomma, 2006; Gowda et al., 2009; Ben et al., 2013; Rosado et al., 2016). Single gene resistance is often complete (i.e., non-host interaction) and pathogen race specific. In comparison, polygenic resistance range from partial to full and is effective against a broad range of isolates within a pathogen population (Michelmore et al., 2013). The ability of the host to rapidly compensate for the lost of xylem vessels due to pathogen compartmentalization by differentiating new functional ones in order to maintain sufficient stem water conductivity may also account for the tolerance to wilt diseases (Talboys, 1972; Fradin and Thomma, 2006).

The spatio-temporal model of compartmentalization of vascular wilt fungi in vessels emerged from observations of annual plants, including tomato (*Solanum lycopersicum*), banana (*Musa* spp.) and cotton (*Gossypium* spp.) (Beckman and Roberts, 1995). In resistant hosts, the sequence includes the entrapment of conidia at the end wall of a vessel (i.e., pit membrane), the secretion of gels that occlude the vessel above the entrapment sites, followed by the development of tyloses that wall off the vessel. Sealing of vessels by gels also favor the local accumulation of antifungal compounds (i.e., phytoalexins, tannins, ROS) next to the entrapment site that can cause inhibition or death of the pathogen (Beckman and Roberts, 1995; Chen et al., 2004; Fradin and Thomma, 2006). In failed compartmentalization such as with tolerant and susceptible hosts, the plug of gels brakes-down under the action of fungal lytic enzyme and/or the sap pressure, before the complete occlusion of vessel lumen with tyloses (Beckman and Roberts, 1995). The conidiospores formed above the entrapment site are further carried in the sap upward to the next vessel end wall eventually leading to systemic infection. In complete occlusions, tylosis walls enter in contact of each other, followed by a maturation process that consists in the inward edification of additional physical layers, such as deposition of cellulo-lignified material and suberin (Rioux et al., 1995). This process aims at reinforcing the inner side of the tylosis membrane, making it a more rigid and impervious wall to pathogen spread. Vascular wilt fungi may be able to escape compartmentalization by the mean of hyphal growth in tyloses-occluded vessels, and subsequently infect new ones (Fradin and Thomma, 2006).

In comparison, the interaction of wilt pathogen with perennial crops has not been studied as extensively as with annual crops. Dutch elm disease, a wilt disease caused by the fungal pathogen *Ophiostoma novo-ulmi* (C.M. Brasier) (Brasier, 1991), is one of the better-known pathosystem. Evidence suggests that the vascular architecture and specifically the diameter of xylem vessel of *Ulmus* genotypes play an important role in the resistance to wilt disease, whereby susceptible hosts presented higher number of vessels of wide diameter than resistant genotypes (Solla and Gil, 2002b; Venturas et al., 2014). More recent observations also suggested that this feature could explain the difference in grapevine cultivars resistance to esca disease (Pouzoulet et al., 2014). The physiological mechanisms linking host resistance to those anatomical differences have remained, however, hypothetical and need further explanation. The goal of this study was to investigate the relevance of plant xylem vessel diameter as a driver of fungal wilt pathogen compartmentalization success or failure in a perennial crop system.

## Materials and Methods

### Evaluation of Grapevine Cultivars Resistance to *P. chlamydospora* in *In Planta* Bioassays

We selected four *V. vinifera* L. cvs based on their different susceptibility levels to esca disease (Feliciano et al., 2004; Bruez et al., 2013; Travadon et al., 2013; Murolo and Romanazzi, 2014). One year-old dormant grape cuttings cvs Merlot (M), Chardonnay (Ch), Cabernet Sauvignon (CS), and Thompson Seedless (TS) were provided by the Foundation Plant Services (Supplementary Table 1). Cuttings were prepared as described by Pouzoulet et al. (2013b) and potted in 4 l pots containing UC-mix soil. Four weeks after potting, homogeneous plants were selected on the basis of height and stem diameter. Inoculations were performed in the dorsal or ventral part of the stem using the drill method. Inoculum was prepared as described by Travadon et al. (2013) using *P. chlamydospora* voucher isolate UCR-Pc4 (Morales-Cruz et al., 2015). Inoculum consisted in one 20 μl drop of sterile 10 mM phosphate buffer (20 μm filtered, pH = 7) containing a total of 1000 *P. chlamydospora* conidia. Control plants received 20 μl drop of 10 mM phosphate buffer. Plants (cultivars and treatments) were organized randomly in a glasshouse (temperature ranged from 21 to 32°C) and watered three times a week. A total of 128 plants were used per year with 32 plants per cultivar (16 inoculated and 16 control plants). Half of the plants (8 inoculated and 8 control plants) were used for the measurement of necrotic lesions and the other half was used for the quantification of fungal DNA in wood fragment above the inoculation site. The experiment was repeated in 2013 and 2014.

Fungal colonization *in planta* was evaluated 10 weeks post-inoculation by measuring necrotic lesion length in the xylem and quantifying the amounts of fungal DNA using a qPCR assay. For the measurement of lesions, stems were longitudinally cut using a scalpel in a plane striking across the wound. Pictures of lesions developed above the inoculation site were taken using a stereo-microscope (M165C, Leica microsystems CMS GmbH, Wetzlar, Germany) and measured using LAS v4.2 software (Leica microsystems CMS GmbH, Wetzlar, Germany). For the quantification of *P. chlamydospora* DNA in xylem fragments, stem samples from the wounded non-inoculated control and *P. chlamydospora* inoculated plants were frozen and subjected to 48 h of lyophilization using Labconco freezone 2.5 l (Kansas City, MO, United States). Samples were cut longitudinally in the plan crossing the bud in order to recover only the half part of stems carrying the infection. For *P. chlamydospora* inoculated plants, two fragment lengths were cut above the inoculated point, the first one from point of inoculation up to 15 mm (level 1: L1) and the second one 15–30 mm (level 2: L2) distant from the inoculation point. Bark and pith were removed from the wood sections using a sterile scalpel. Samples were ground with a mixer mill (MM 400, Retsch GmbH, Haan, Germany) and DNA was extracted as described previously (Pouzoulet et al., 2013b). Concentration of total DNA extracted was determined using a Qubit^TM^ fluorometer (Invitrogen, Carlsbad, CA, United States) and the Quant-it dsDNA high-sensitivity reagent (Invitrogen) according to the manufacturer protocol. The qPCR reactions proceeded in a final volume of 25 μl, and reaction mixtures contained 12.5 μl of 2X SYBR^®^ Green Quantitect^®^ Master Mix (Qiagen, Venlo, Netherlands). Primers PchQF (5′-CTCTGGTGTGTAAGTTCAATCGACTC-3′)/PchQR (5′-CCATTGTAGCTGTTCCAGATCAG-3′) were used at a final concentration of 0.5 μM. Two μl of DNA template were used per reaction. Experiments were conducted with a CFX96 Real-Time PCR cycler using CFX manager software v3.1 (Bio-Rad, Irvine, CA, United States). The cycling program consisted of (1) an initial denaturation step at 95°C for 15 min, (2) 40 cycles of 15 s at 95°C (for denaturation) followed by 45 s at 62°C (for both annealing and extension), and (3) an additional melting analysis of 40 min from 60 to 95°C. Preparation and use of standard solutions for the absolute quantification of *P. chlamydospora* isolate UCR-Pc4 DNA was done as previously described (Pouzoulet et al., 2013b). Average of absolute amounts of *P. chlamydospora* DNA determined by qPCR in three independent technical replicates were standardized on the amounts of input DNA and used for further statistical analysis. The qPCR values observed in levels L1 and L2, cultivars and years were subjected to an ANOVA using SAS Proc Mixed (version 9.4; SAS Institute, Cary, NC, United States) followed by multiple comparisons of means using Fisher’s protected least significant difference test.

### Assessment of Xylem Vessel Diameter in Grapevine Cultivars and Correlation with *P. chlamydospora* Resistance

An automated detection analysis of vessels and measurement of their arithmetic diameter were performed on stem cross-sections as described by Scholz et al. (2013). Six internode segments (three stems from two different mother-vines) of 8 to 10 mm of diameter and 100 to 120 mm in length were sampled within cutting bundles of each cultivar (Supplementary Table 1). A 10 mm long fragment was sampled in the middle part of the each internode. Samples were fixed in ethyl-alcohol 80% at 4°C during 48 h, after which ethyl-alcohol solution was replaced and samples were kept at 4°C. High definition micrographs (430 pixels mm^-1^) covering a quarter of each Toluidine O stained stem cross sections (70 μm thick) were obtained as described by Pouzoulet et al. (2014). For each stems, number of vessels and vessel density (vessel count mm^-2^) were determined from six consecutive fascicular portions within the dorsal or ventral area using ImageJ v1.48^1^. Arithmetic vessel diameter was computed according to Scholz et al. (2013) and vessel distribution per class of diameter was determined using Excel 2010 (Microsoft Corporation, Redmond, WA, United States). The density of vessels for different classes of diameter (20 μm steps) observed in the four cultivars studied was subjected to an ANOVA using SAS Proc Mixed (version 9.4; SAS Institute, Cary, NC, United States) followed by multiple comparisons of means using Fisher’s protected least significant difference test.

### Histological Characterization of *P. chlamydospora* Compartmentalization in Grapevine Xylem

Shoots of 1 year-old grapevine cv CS were potted in 8 l container and grown in glasshouse for 2 months. Soil was watered to saturation every 2 days during the period of the experiment. Plants developed 4 m long shoots with a basal diameter of 8 to 10 mm. Shoots were wounded in dorsal area using a drill method (2 mm of diameter) at the third node. Five plants were inoculated with a 20 μm filtered solution of phosphate buffer (pH = 7), and the five plants were inoculated with a suspension of *P. chlamydospora* spore as described previously. Eight weeks after treatment, internode fragments above the inoculation wound were sampled and fixed in FAA solution (Formaldehyde, Acetic acid, Ethyl Alcohol; 5/5/9) for 48 h at 4°C, then rinsed and conserved in 80% Ethyl alcohol at 4°C. Sections (70 μm thick) were obtained as previously described. Staining of specimen with Toluidine O (0.05%, pH = 4.3, Sigma–Aldrich, St Louis, MO, United States), IKI (potassium iodide-iodine, Ricca Chemical Company, Arlington, TX, United States), ruthenium red (0.005%, Sigma–Aldrich), Phloroglucinol/HCl (Sigma–Aldrich), and Sudan IV (0.01% in 70% ethyl-alcohol, Sigma–Aldrich) were adapted from Ruzin (1999). Light microscopy works were done using a Leica DM 4000 upright microscope (Leica Microsystems CMS GmbH, Wetzlar, Germany). Epifluorescent micrographs were obtained using an inverted epifluorescent microscope (Arcturus XT, Life Technologies, Carlsbad, CA, United States) by overlaying micrographs from the ‘UV’ (excitation: 325–375 nm, emission: >420 nm) and ‘green’ filters (excitation: 510–560 nm, emission: >590 nm) with ImageJ v1.48. For the co-visualization of fungal hyphae and plant material by epifluorescent and confocal microscopy, plant sections were placed in a 2 ml plastic tube containing 1 M potassium hydroxide and incubated 1 h in boiling water. Sections were then rinsed three times during 10 min in deionized water. Sections were stained using an aqueous solution of Auramine O (0.1%, Sigma–Aldrich) for 5 min for the staining of plant material followed by 20 min of staining with Calcofluor M2R White (Sigma–Aldrich) for the labeling of the fungal cell wall. Sections were briefly rinsed and mounted in deionized water. Epifluorescent and confocal microscopy works were realized at the Microscopy Core/Center for Plant Cell Biology at the Institute for Integrative Genome Biology at the University of California, Riverside. Epifluorescence micrographs were obtained with an epifluorescent microscope Arcturus XT^TM^ using UV filter. Confocal micrographs were obtained using a Leica SP2 (Leica microsystems CMS GmbH, Wetzlar, Germany) equipped for UV microscopy. Two channels were used, one for the recovery of calcofluor M2R white related signal (excitation = 364 nm, detection = 410–505 nm, displayed in green), and one for the recovery of the Auramine O related signal (excitation = 488 nm, detection = 505–590 nm, displayed in magenta). Acquisition of channels was done in sequence. Acquisition of *Z*-stacks varying from 50 to 100 μm of depth were done using a water immersion objective (20×), micrographs being taken approximately every 1 μm. Z-projections of micrographs were done with ImageJ v1.48 using the standard deviation method. Both inoculations and microscopic observations were replicated twice on different sets of plants, and an interval of 6 weeks.

### Modeling of Xylem Vessel Occlusion with Tylosis

We evaluated the impact of xylem morphology on quantity of tyloses during vessel occlusion. Five cuttings from all four grapevine cvs were wounded and grown for 10 weeks as described previously. Xylem segments above the wound were sampled and fixed in FAA and 70 μm cross-sections of xylem region located 5 mm above the wound were obtained as described previously. Sections were dehydrated in 100% ethanol, dried at room temperature for 15 min and observed using a tabletop scanning electron microscope (Hitachi TM-1000, Tokyo, Japan). The arithmetic vessel diameter, the number of tyloses (*N*_tyl_) and the outer tylosis surface length (OTSL) were measured with ImageJ v1.48 software. Preliminary analyses indicated that the relationships between the vessel diameter (*D*, μm) and the number of tylosis, and the vessel diameter and the OTSL (μm) was of non-linear nature, and could be described with an exponential curve (Eqn. 1):

(1) $$\mathrm{Ntyl}\mathrm{or}OTSL=\alpha D^{\;\beta }$$

where α and β were the model parameters. By applying a natural logarithm transformation to Eqn. 1 we obtained the following equation (Eqn. 2):

(2) ln (Ntyl) or ln (OTSL)=ln(α)+β×ln(D)

It was possible to parameterize the model with an ordinary least square (OLS) approach. Eqn. 2 was parameterized by Generalized Reduced Gradient optimization (Lasdon et al., 1978), using the Excel 2010 Solver tool (Frontline Systems, Incline Village, NV, United States). Eqn. 2 described the global (i.e., for all four cvs) relationships. The robustness of the global model was tested using a leave-one-group (i.e., cvs)-out cross-validation (Martens and Dardenne, 1998). Note that curves presented correspond to the back-transformed data.

## Results

### Evaluation of Grapevine Cultivars Resistance to *P. chlamydospora* in *In Planta* Bioassays

**FIGURE 1:**
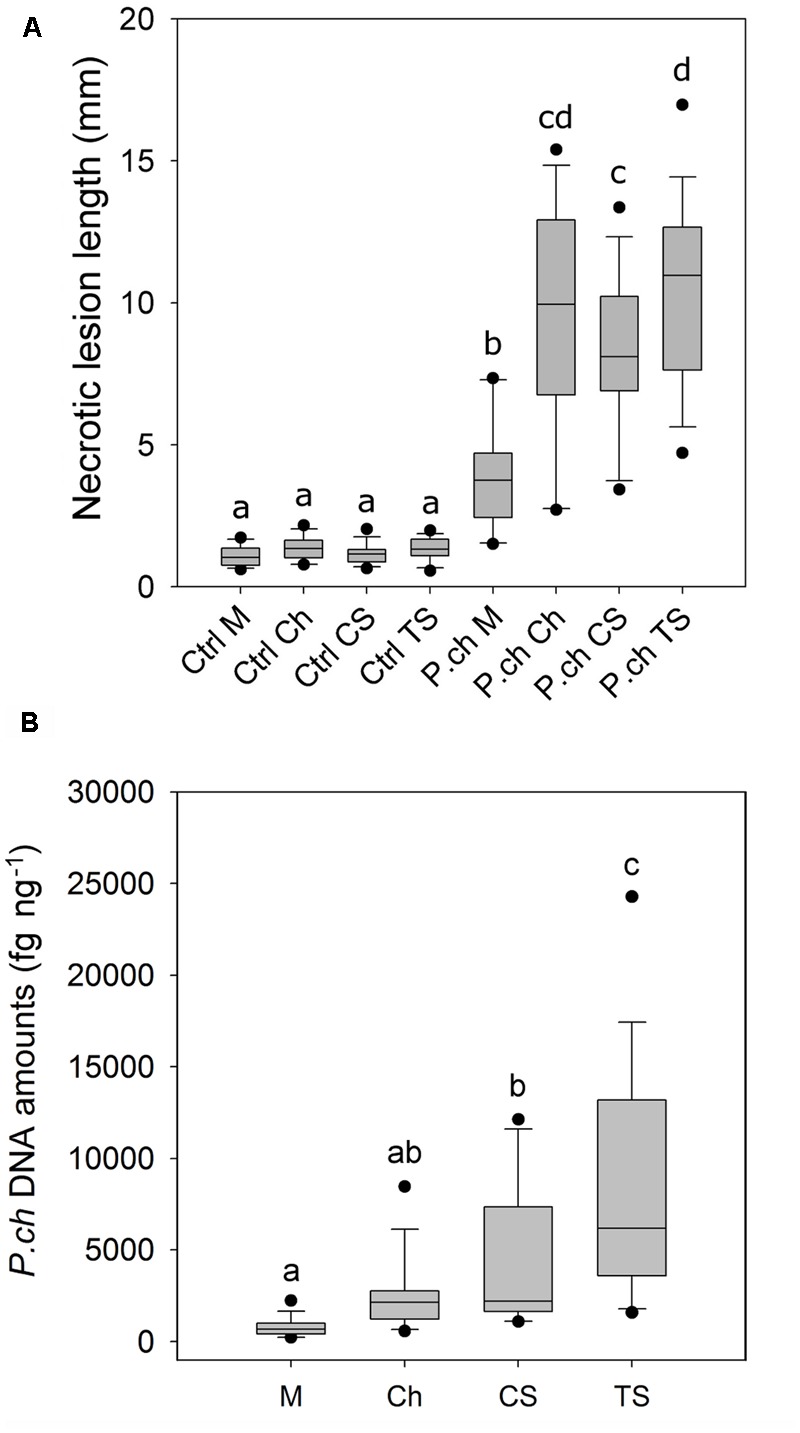
Level of susceptibility of *Vitis vinifera* cultivars to *P. chlamydospora* (*P. ch*) as expressed by wood necrotic lesion length and fungal DNA amounts in *in planta* bioassays. **(A)** Wood necrotic lesion length that developed above the inoculation point. **(B)** Amount of *P. chlamydospora* DNA in xylem tissue fragments above and distant from the inoculation point (L2; 15–30 mm). Pooled data from 2013 and 2014 trials are shown. Different letters indicate statistically significant difference (*P* < 0.05). Detailed outputs of the statistical analyses of data are provided in **Table 1** and Supplementary Tables 2, 3. M, Merlot; Ch, Chardonnay; CS, Cabernet Sauvignon; TS, Thompson Seedless.

Necrotic lesion length and fungal DNA amount in wood tissue were used as criteria to assess the level of resistance of grape cultivars to *P. chlamydospora*. There were no significant interactions between cultivar and year for the necrotic lesion length (**Table 1**) and data from both years were pooled together (**Figure 1A**). After 10 weeks of incubation, *P. chlamydospora* inoculated plants developed wood necrotic lesions significantly longer than their respective controls for the four cultivars tested. *Post hoc* analysis classified all mock-inoculated plants in a same statistical group, while inoculated plants were classified in different groups (**Figure 1A**). Among all four cultivars, wood necrotic lesions that developed in M were significantly smaller than the other cultivars and were classified on this basis as the most resistant to *P. chlamydospora*. In contrast, TS developed the longest necrotic lesions and was classified as the most susceptible cultivar. CS showed intermediate lesion length and differ significantly from both M and TS. Ch also showed an intermediate susceptibility and differed significantly from M, but not from either CS or TS (**Figure 1A**).

**Table 1:** Analyses of variance (ANOVA) showing the effect of inoculation, year, grape cultivar and their interactions for the *in planta* resistance bioassay.

Effect	Pr > F		
	Necrotic lesion	*P. chlamydospora*	*P. chlamydospora*
	length (mm)	*DNA amount (L1)*	*DNA amount (L2)*
Inoculation	<0.001^∗∗∗^	n/a	n/a
Year	0.23	<0.001^∗∗∗^	<0.001^∗∗∗^
Cultivar	<0.001^∗∗∗^	<0.001^∗∗∗^	<0.001^∗∗∗^
Inoculation^∗^Year	0.87	n/a	n/a
Inoculation^∗^Cultivar	<0.001^∗∗∗^	n/a	n/a
Year^∗^Cultivar	0.11	<0.001^∗∗∗^	<0.005^∗∗∗^

*^∗∗∗^Significant at the 0.001 probability level. ^†^NS, not significant at the 0.05 probability level. ^‡^n/a, not applicable.*

*The level of cultivar resistance was assessed by both the necrotic lesion length that developed upward from the inoculation point and the qPCR quantification of *P. chlamydospora* DNA at two lengths above the inoculation point (L1 = 0–15 mm and L2 = 15–30 mm) (*n* = 128 for necrotic lesion; *n* = 64 for *P. chlamydospora* DNA amounts due to the exclusion of control plants from the analysis, i.e., absence of *P. chlamydospora* DNA).*

*Phaeomoniella chlamydospora* DNA amount was measured in wood tissue close (L1) and distant (L2) from the inoculation point (**Figure 1B**). Fungal DNA was consistently detected in xylem tissue the most distant from the inoculation point (L2) for all four cultivars despite the fact that these fragments were mostly asymptomatic (i.e., no wood necrotic lesions beyond 15 mm; **Figure 1A**). L1 and L2 were analyzed separately for statistical purposes. In addition, only values from inoculated plants were considered for its statistical analysis because no *P. chlamydospora* DNA was detected in control plants. For L1, statistical analysis of *P. chlamydospora* DNA amount detected a significant interaction between cultivar and year (**Table 1** and Supplementary Table 2). Differences between the 2 years were observed with overall lower DNA amounts in 2014 vs. 2013. Statistical classification for grape cultivars was not consistent across the 2 years. For L2, statistical analyses of the *P. chlamydospora* DNA amounts also revealed an interaction between cultivar and year (**Table 1** and Supplementary Table 3). As with L1, our data showed overall lower fungal DNA amount measured in wood for 2014 compared to 2013 but with a similar trend in both year, a better statistical segregation of cultivars being observed in 2013 than 2014 (Supplementary Table 3). Finally, L2 pooled data from 2013 and 2014 separated M, CS and TS in three groups of resistance, M being the most resistant, followed by CS and TS, the most susceptible, whereas Ch belonged to an intermediate group (**Figure 1B**). Observations from wood necrotic lesions and amounts of *P. chlamydospora* DNA in L2 were found to correlate significantly (*R*^2^ = 0.60, *p* = 0.025, *n* = 8), thus supporting overall similar conclusion on the level of resistance of grape cultivars in our bioassays.

### Assessment of Xylem Vessel Diameter in Grapevine Cultivars and Correlation with *P. chlamydospora* Resistance

**FIGURE 2:**
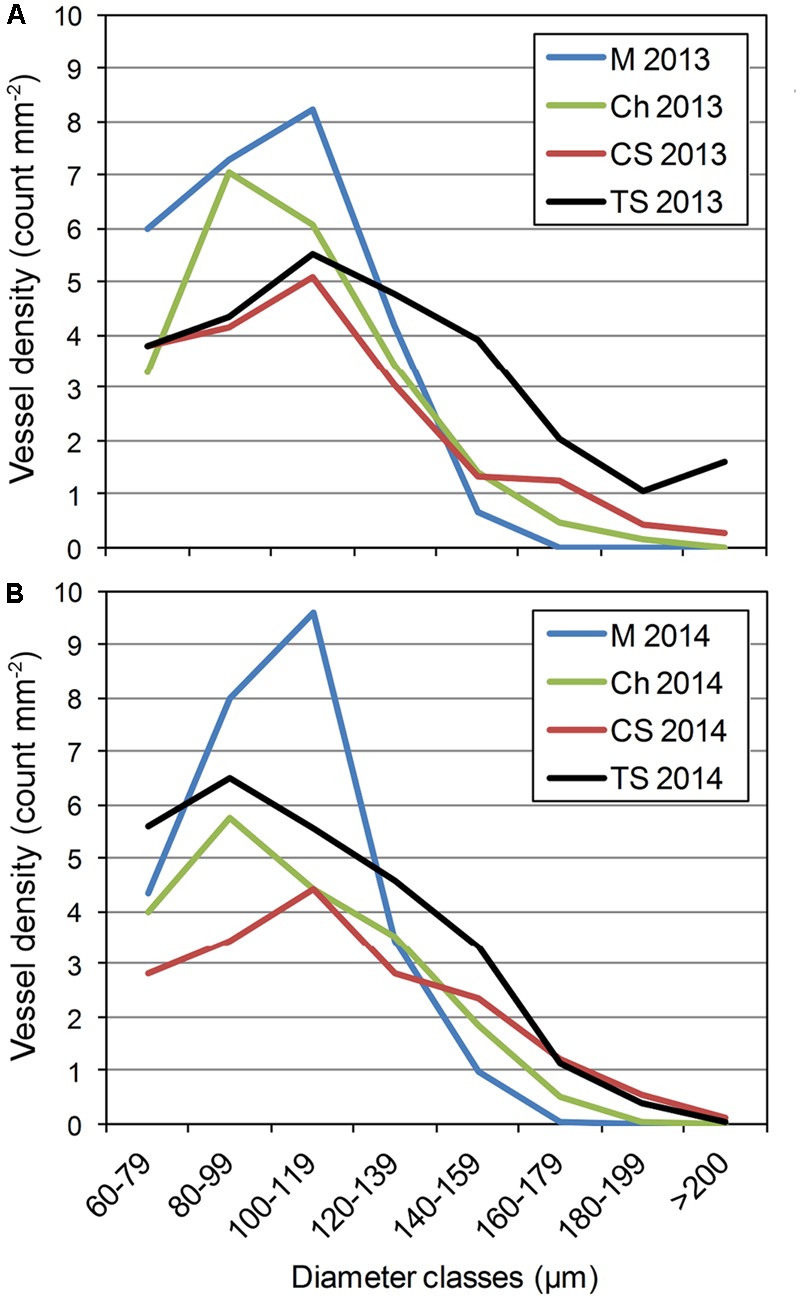
Distribution of xylem vessel diameter observed in the stem of *V. vinifera* L. cvs Merlot (M), Chardonnay (Ch), Cabernet Sauvignon (CS), and Thompson Seedless (TS) in **(A)** 2013 and **(B)** 2014. Please see Supplementary Tables 4, 5 for further statistical analysis of distributions.

Wood morphology was analyzed on the dorsal or ventral area of grapevine stems, because this is where *P. chlamydospora* inoculations were performed. Vessel distributions of the plant materials were overall consistent between 2013 and 2014 (**Figure 2**). For the number of vessels per diameter class, ANOVA shows a significant effect of cultivars for many diameter classes (e.g., 80–99, 100–119, 140–159, 160–1879, and 180–199 μm), but also some interactions between cultivar and year (i.e., 60–79, *d* > 200; Supplementary Tables 4, 5). Our results indicated that M, the most resistant cv, displayed the highest number of narrow diameter vessels (i.e., from 80 to 119 μm), and the lowest number of wide diameter vessels (i.e., above 120 μm). In contrast TS, the most susceptible cv, displayed the highest number of wide diameter vessels (**Figure 2**). Ch and CS, of intermediate resistance, displayed an intermediate number of wide diameter vessels (**Figure 2**).

To confirm that cv resistance was related to xylem morphology, regressions were calculated between the density of vessel superior to a fixed diameter (e.g., *d* > 100, *d* > 120, ..., *d* > 200) and *P. chlamydospora* DNA amount (**Table 2**). We found significant correlations between the amount of *P. chlamydospora* DNA (Log_e_ transformed) from the L2 and the number of vessels superior to 120, 140, 160, and 180 μm diameter per mm^2^ (**Table 2**). These results supported our hypothesis, whereby the diameter of xylem vessels affects *P. chlamydospora* compartmentalization outcome in grapevine and in turn cultivar resistance.

**Table 2:** Correlation between the degree of resistance of *Vitis vinifera* cultivars and the density of wide diameter xylem vessels.

Vessel	Necrotic lesion	ln	ln
diameter	length	(*P. chlamydospora*)	(*P. chlamydospora*
class		DNA amount in L1)	DNA amount in L2)
*d* > 60	0.029	0.11	0.0037
*d* > 80	0.01	0.11	0.0025
*d* > 100	0.02	0.001	0.19
*d* > 120	0.33	0.15	0.63^∗^
*d* > 140	0.38	0.18	0.73^∗∗^
*d* > 160	0.29	0.26	0.74^∗∗^
*d* > 180	0.16	0.19	0.59^∗^
*d* > 200	0.08	0.15	0.47

*^∗^Significant at the 0.05 probability level. ^∗∗^Significant at the 0.01 probability level.*

*The table displays the coefficient of determination (*R*^2^) of linear regressions calculated between the density of vessels (number of vessel per mm^2^) superior to a defined diameter and the mean of necrotic lesion length, and the natural logarithms of the means of *P. chlamydospora* DNA amount in xylem fragments within the first 15 mm (L1) and within 15–30 mm (L2) above the inoculation point (*n* = 8).*

### Histological Characterization of *P. chlamydospora* Compartmentalization in Grapevine Xylem

**FIGURE 3:**
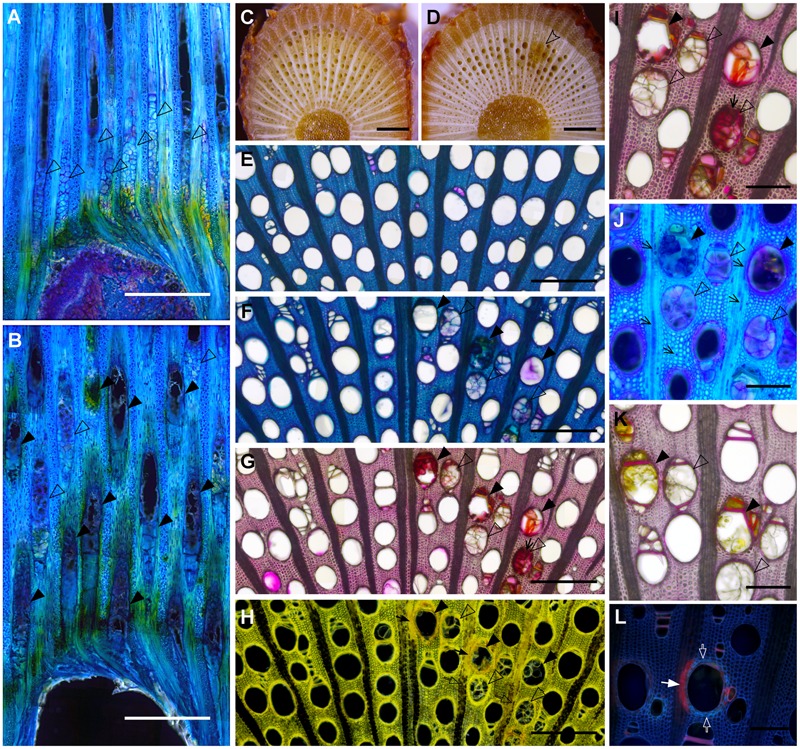
Histological characterization of the compartmentalization process of *P. chlamydospora* in *V. vinifera* L. cv Cabernet Sauvignon stem. **(A,B)** Micrographs of tangential sections of Mock **(A)** and *P. chlamydospora* inoculated **(B)** plants showing the plant response right above the inoculation point (Toluidine O, dark field/phase contrast) 2 months post-inoculation. **(A)** Note the presence of vessel fully occluded by tyloses (hollow black arrowheads) about 1 mm above the wound in control plants and the presence of scar tissue at the inoculation point. **(B)** Note the presence of both fully occluded (hollow black arrowheads) and partially occluded vessels (solid black arrowhead) above the wound in *P. chlamydospora* inoculated plants and the absence of scar tissue at the inoculation point. **(C,D)** Pictures showing macroscopic phenotype 2 cm above the point of inoculation in Mock **(C)** and *P. chlamydospora* inoculated shoots **(D)**. Note the presence of typical wood necrotic lesion (hollow arrowhead) caused by *P. chlamydospora* **(D)** while no symptoms are observed in Mock inoculated plants **(C)**. **(E)** Micrograph of Mock inoculated stem in cross section 2 cm above the point of inoculation (bright field, toluidine O). **(F–L)** Micrograph of *P. chlamydospora* inoculated stem in cross section 2 cm above the point of inoculation **(F**, toluidine O, bright field; **G** and **I**, ruthenium red, bright field; **H**, iodine potassium iodide, dark field/phase contrast; **J**, toluidine O, dark field/phase contrast; **K**, phloroglucinol/HCl, bright field; **L**, sudan IV, epifluorescence UV). Note the presence of partially occluded vessels (solid black arrowheads) and fully occluded vessel (hollow black arrowheads) in the necrotic area of the xylem. **(G,I)** Large field **(G)** and close view **(I)** of section stained for the presence of pectin (ruthenium red). Note the presence of deep pink coloration that developed in partially occluded and some of the fully occluded vessels (black arrows) indicating the presence of gels rich in pectin. **(H)** Cross-section stain for the presence of starch (iodine potassium iodide). Note the complete depletion (black arrows) of starch (stained in black) in ray parenchyma cells next to partially occluded vessel (solid black arrowheads), while only partial depletion (hollow black arrows) occurs in ray parenchyma cells next to fully occluded vessels (hollow black arrowheads). **(J)** Micrographs showing the accumulation of putative non-structural phenolic compounds (staining in deep dark blue) in the lumen of ray parenchyma cells and some living fibers (black arrows) stained by the toluidine O. **(K)** Cross-section stained for the presence of lignin (phloroglucinol/HCl). Note the absence of change in purple color intensity in the wall of ray parenchyma cells and fiber surrounding partially and fully occluded vessel, indicating no substantial accumulation of structural lignin. **(L)** Cross-section stained for the presence of suberin (Sudan IV). The vessel presented in the center of the picture is partially occluded. Note the presence of enhance autofluorescence in the cell wall of paratracheal parenchyma cells and fibers surrounding the vessel (hollow white arrow), that could be attributed to the infusion of non-structural phenolics. Also note the presence of red fluorescence indicating a deposition of suberin in the wall of ray parenchyma cells next to the vessel (solid white arrow). **(A–D)** scale bar = 1000 μm. **(E–H)** scale bar = 500 μm. **(I–L)** scale bar = 200 μm.

To better understand how vessel diameter could affect the level of resistance to *P. chlamydospora* in grapevine, we first characterized the plant response to infection and the fungal colonization strategy in a single cultivar. CS was used due to its intermediate level of resistance. Observation of tangential sections next to the point of inoculation in control plants showed a mass of scar tissue (a.k.a. bark ridge) that filled the wound and xylem vessel occlusion with tyloses that extended about 1 mm above the wound (**Figure 3A**). In contrast, no scar tissue could be found in *P. chlamydospora* inoculated plants and occlusion of vessels extended far beyond the inoculation point (**Figure 3B**). Shoot cross-sections 20 mm above the inoculation point showed typical necrotic wood lesions caused by *P. chlamydospora* (**Figures 3D,F**) while absent in mock-inoculated plants (**Figures 3C,E**). Symptomatic area displayed occluded vessels, either partially or completely, with tyloses and gels (**Figures 3F–H**). The use of Ruthenium Red revealed staining mostly of partially occluded vessels, although occasionally some reactions occurred with apparently fully occluded vessels (**Figures 3G,I**), suggesting the presence of immature tyloses and/or pockets of pectin-rich gels. The use of IKI stain revealed that the response to infection was associated with a depletion of starch storage in ray parenchyma cells next to occluded vessels (**Figure 3H**). However, starch depletion was only partial next to fully occluded vessels, whereas it was much more severe next to vessels partially occluded. Staining with Toluidine O indicated the presence of non-structural phenolics in the lumen of ray parenchyma and living fiber for both mock and *P. chlamydospora* inoculated plants (**Figures 3A,B,J**). However, the accumulation of phenolics often observed in cells next to partially occluded vessels suggests a local response to infection (**Figure 3J**). Characterization of cell wall modifications occurring in symptomatic area using the Mäule test indicated no noticeable change in structural lignin (**Figure 3K**). The use of Sudan IV and UV epifluorescence microscopy, however, revealed the deposition of suberin in ray parenchyma cells next to partially occluded vessels, indicating that a second line of local defense took place in tissue surrounding infected vessels (**Figure 3L**).

**FIGURE 4:**
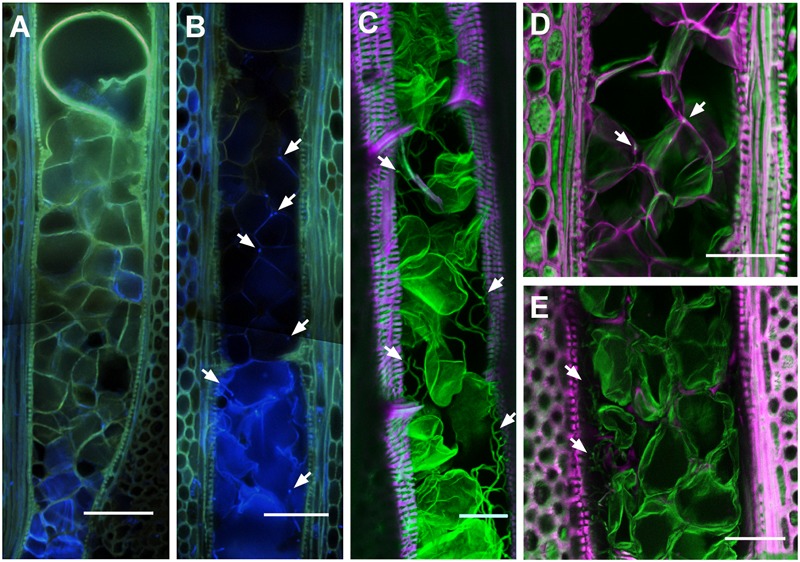
Localization of *Phaeomoniella chlamydospora* hyphal structures in the xylem of infected *V. vinifera* L. cv Cabernet Sauvignon stem. **(A,B)** Epifluorescent micrograph showing tangential section of Mock **(A)** and *P. chlamydospora* inoculated **(B)** plants within the first mm above the point of inoculation (Auramine O, Calcofluor White M2R; UV illumination). **(A)** Note the positive staining of the cell wall of tyloses with Auramine O (greenish) indicating the accumulation of lignin and/or suberin. **(B)** Note the presence of damaged tyloses in the lower part of the vessels, not observed in the Mock inoculated plants. Also note the presence of fungal structures (bright blue spots, white arrows) associated with damage tyloses in the lower part of vessel, and along the tyloses cell wall surface in the upper part of the vessel (white arrows). Confocal micrographs of longitudinal **(C–E)** from the necrotic lesion of xylem tissue infected with *P. chlamydospora*. Samples were stained with Auramine O for the visualization of cellulo-lignified and suberized plant structures (excitation: 488 nm, emission: 505–590 nm; displayed in magenta) and calcofluor M2R white for the visualization non-lignified plant structures and fungal hyphae (excitation: 364 nm, emission: 410–505 nm; displayed in green). **(C)** Micrographs of a large vessel heavily colonized by fungal structures (white arrows). Note the green signal of the cell wall of tyloses indicating its incomplete maturation (i.e., absence of lignin and suberin). **(D)** Micrograph showing fungal structures (white arrows) at the intersection of tyloses and along the inter-tyloses cell wall boundary in a vessel fully occluded with mature tyloses (white arrows). **(E)** Micrograph showing a dense network of fungal structures (white arrows) in spaces between the tyloses and the wall of large vessels. **(A,B)** Scale bars = 100 μm. **(C–E)** Scale bars = 50 μm.

To better understand the colonization strategy of *P. chlamydospora* in vessels, the fungus was located *in situ* within symptomatic area of the xylem of CS shoots. For the co-visualization of *P. chlamydospora* hyphae and plant material by epifluorescent and confocal microscopy, best results were obtained using Auramine O (for the labeling of the plant material) counter stained with Calcofluor White M2R (for labeling of the fungal cell wall) (**Figure 4**). Observation of mock-inoculated plants showed no fungal structures in the xylem vessels (**Figure 4A**) indicating that those observed in the inoculated plants likely belonged to *P. chlamydospora* (**Figures 4B–E**). In vessels next to the point of inoculation, *P. chlamydospora* was detected in association with both damaged and intact tyloses (**Figure 4B**). In some vessels of wide diameter where occlusion failed, a dense network of hyphae was often observed in the xylem lumen along the vessel wall (**Figure 4C**). In other large vessels where occlusion was apparently successfully, the presence of *P. chlamydospora* hyphae was associated with deteriorated clusters of tyloses, where it progressed in between the network of mature tyloses walls and also between tyloses and the vessel wall (**Figures 4D,E**).

### Modeling of Xylem Vessel Occlusion with Tylosis

**FIGURE 5:**
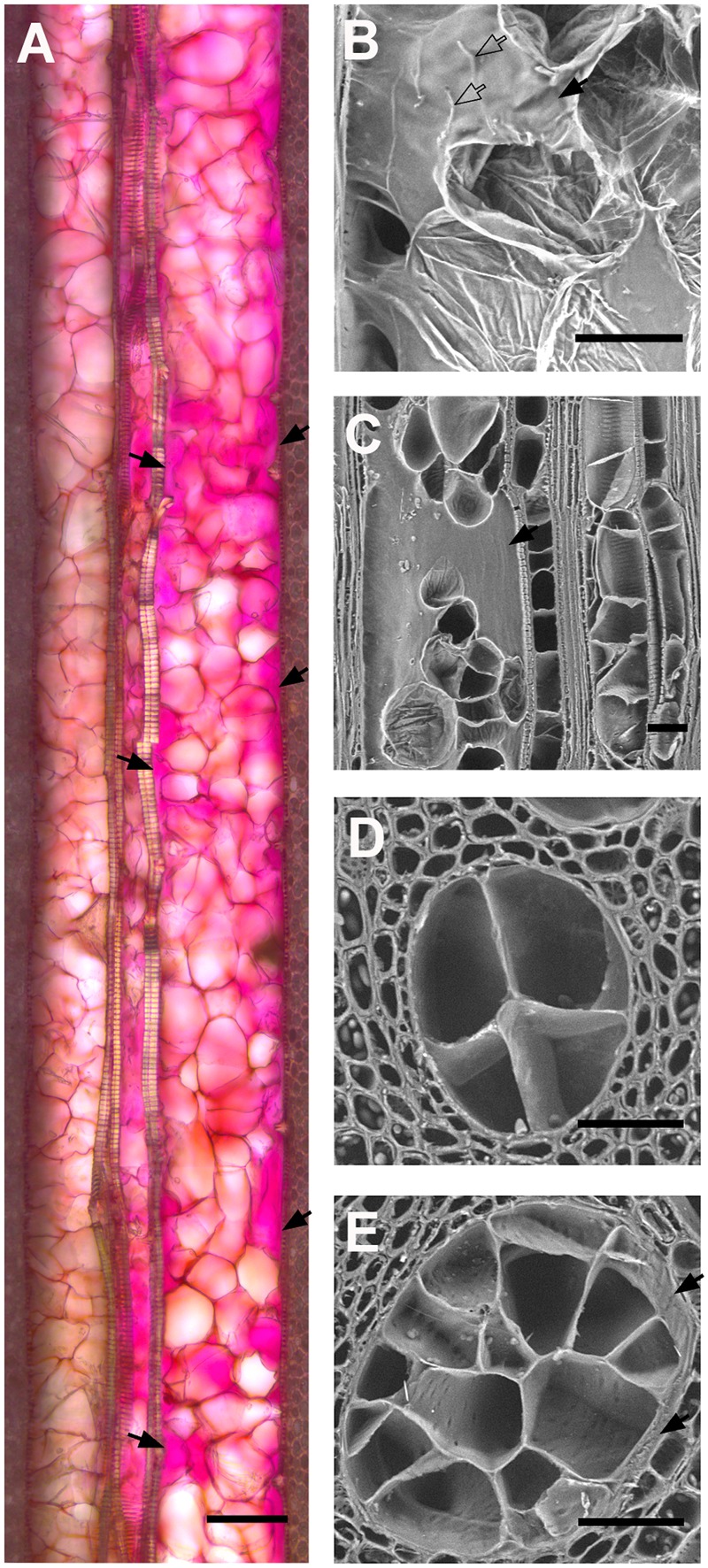
Effect of vessel diameter on the quality of vessel occlusion with tyloses in four *V. vinifera* L. cultivars. **(A)** Micrograph of longitudinal section of occluded vessels found in *Phaeomoniella chlamydospora* inoculated grape cv Cabernet Sauvignon (bright field, ruthenium red). Note the difference in the diameter of the vessel on the left side (about 90 μm of diameter) and the vessel on the right side (about 150 μm of diameter). Note the compact network of tyloses that walls off the narrowest vessel while large pockets of gels (solid black arrows) are present in the wider vessel. **(B,C)** Scanning electron micrograph of a longitudinal section of *P. chlamydospora* infected grapevine cv Thompson Seedless. Note the presence of a large pocket of gels observed in occluded vessel. **(D,E)** Scanning electron micrographs of occluded vessels of various diameters as observed in the stem of grapevine cv Thompson Seedless infected with *P. chlamydospora*. Note the increasing number of tyloses occluding cross-sectional area of vessels with the increase of vessel diameter. **(A,B)** Scale bar = 100 μm. **(C–E)** Scale bar = 50 μm.

**FIGURE 6:**
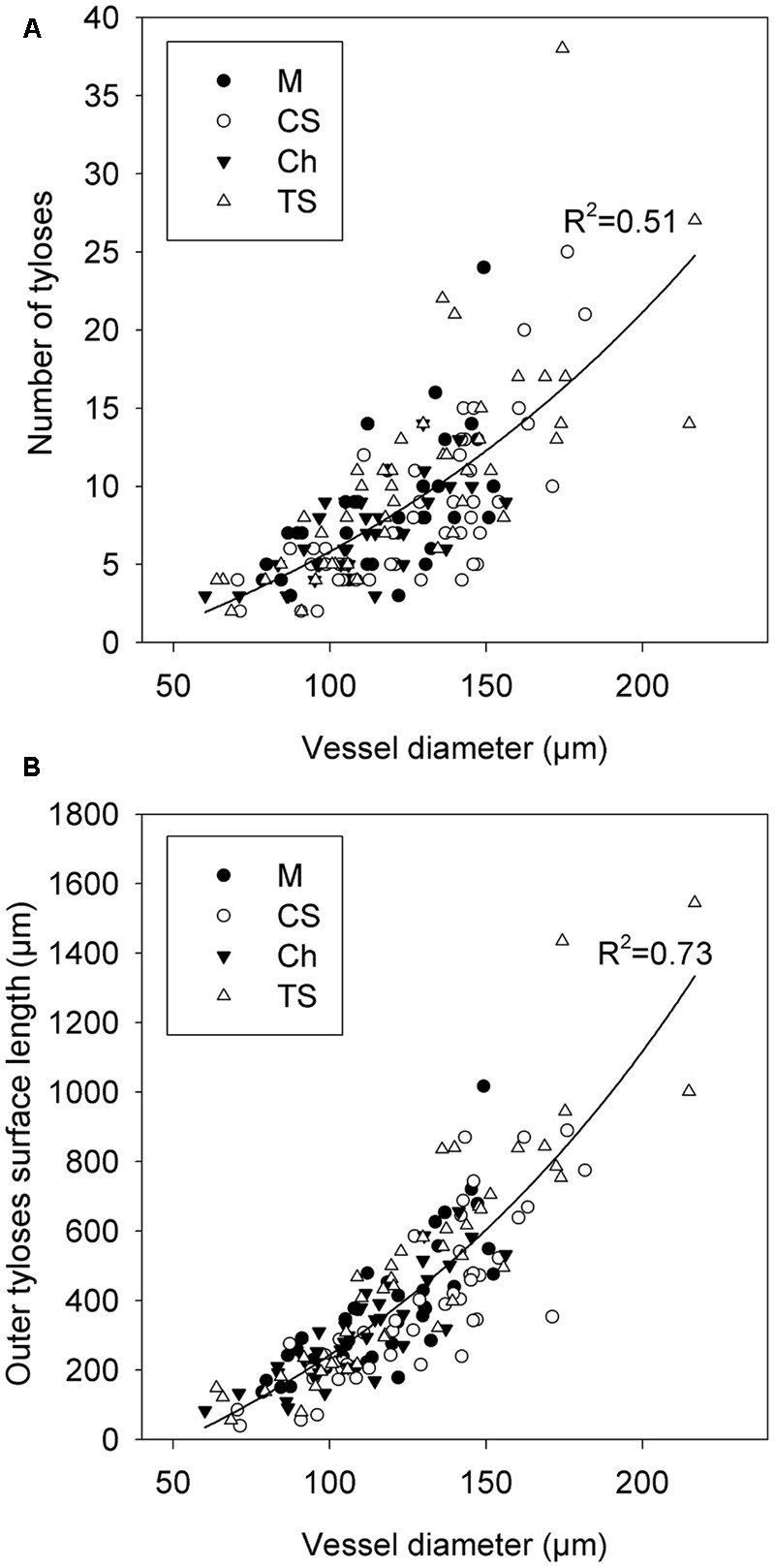
Modeling of the effect of xylem vessel diameter on the quality of vessel occlusion with tyloses across four *V. vinifera* L. cultivars. **(A)** Graphic showing the relation between the arithmetic diameter of vessel and the number of tyloses occluding the cross-sectional area of vessel. **(B)** Graphic showing the relation between the arithmetic diameter of vessel and the outer tyloses surface length (OTSL). Regression curve in **(A,B)** represent the general model developed with data collected from the grapevine cvs Merlot (M), Chardonnay (Ch), Cabernet Sauvignon (CS), and Thompson Seedless (TS).

We first investigated how the diameter of vessels could impact the quantity of tyloses that were formed during occlusion in grapevine cvs. We observed that the occlusion in narrow xylem vessels featured a compact cluster of tyloses whereas in wide vessels a loose cluster of tyloses with pockets of pectic gels was often observed (**Figures 5A–C**). Scanning electron microscopy images revealed that the number of tyloses and the apparent tyloses cell wall surface increased with vessel diameter and this was consistent for all grape cultivars (**Figures 5D,E**). These observations were quantified and our data showed a strong positive correlation between the numbers of tyloses found in vessel cross-sectional area with the increase of xylem vessel diameter (**Figure 6A**). A strict correlation (*R*^2^ = 0.73) was also found between the diameter of vessels and the outer tylosis surface length (OTSL) (**Figure 6B**). Really high dependence (*R*^2^ = 0.97) was found between the number of tylosis and the OTSL. To determine whether these relations could differ amongst grape cultivars, a leave-one-group-out cross-validation scheme was performed. For every combination of grape cultivars, the regressions calculated with the data collected from three grape cultivars were found to explain with confidence (*P* < 0.05) the data observed on the fourth one. These observations confirmed that the influence of the diameter on the resulting tyloses network occluding a vessel was a commonly shared physiological process among the four cultivars studied.

## Discussion

This study provides insightful information about the mechanism of compartmentalization of a wilt pathogen in a woody perennial crop and identifies xylem anatomy as a key determinant of disease resistance. In woody plants compartmentalization is instrumental to wall-off vascular pathogens and ensure that the integrity and physiological functions of the xylem, phloem, and cambium are maintained (Pearce, 1996; Shigo and Marx, 1977; Morris et al., 2016). Vessel occlusion is a critical component of the compartmentalization model for xylem dwelling wilt pathogens because it impairs their movement *in planta* (Beckman and Roberts, 1995; Pearce, 1996; Sun et al., 2013). It is a conserved mechanism among plants and is induced by ethylene production as a response to stress such as wounding or pathogen infection (Beckman and Roberts, 1995; Sun et al., 2007; De Micco et al., 2016). Our data supported these observations and also showed that tylosis formation was greatly stimulated when wounding was combined with pathogen inoculation. In addition, we confirmed that grape genotypes possess different xylem vessel diameter size range (Pouzoulet et al., 2014). The role of xylem architecture and morphology in the resistance to vascular wilt diseases in perennial plants was only previously reported for Dutch elm disease (DED). Similarly to our results, data showed that a high proportion of vessels above 100 μm in diameter negatively correlated with host resistance to DED (Solla and Gil, 2002b; Venturas et al., 2014). Our data showed that the density of vessels superior to 120 μm in diameter significantly correlated with the amount of *P. chlamydospora* DNA in xylem fragment remote from the inoculation site. These observations supported that a less efficient restriction of *P. chlamydospora* movement was achieved with grape cv that harbored proportionally more vessels of wide diameter such as Thompson seedless than with cv such as Merlot that displayed proportionally more vessels of narrow diameter. Conclusions of our resistance *in planta* bioassays corroborated with previous reports whereby Merlot has been accepted as a relatively resistant cv to esca whereas Thompson seedless is in contrast much more susceptible to the disease (Feliciano et al., 2004; Bruez et al., 2013; Murolo and Romanazzi, 2014).

We provide evidence that the occlusion processes taking place in vessels of wide diameter creates a favorable environment for *P. chlamydospora* to grow, with an increased number of routes to escape the compartmentalization process than in narrow diameter vessels. Tyloses are out growth of parenchyma cells pit membranes and as such are mainly composed of pectin rich materials (Rioux et al., 1998). Once tylosis fill the xylem lumen space and enter in contact with each other their cell wall undergoes a maturation process and becomes a rigid wall with inner deposition of cellulose, lignin and suberin (Rioux et al., 1995, 1998; Sun et al., 2013; De Micco et al., 2016). Narrow spaces remaining along the inter-tylosis boundary network become filled with proteinaceous gels, which assure the mechanical cohesion of tyloses clusters (Rioux et al., 1998). Once compartmentalized, wilt pathogens need to actively grow through the maze of tyloses by breaking down wall polymers via enzymatic activities. In addition, vascular wilt fungi require breaking down pit membranes to grow out of xylem vessels and colonize neighboring functional vessels. Although intervessel pit membrane has been considered pectin rich, there is no supporting evidence that this compound is actually present in mature vessels of perennial angiosperms (Plavcova and Hacke, 2011; Kim and Daniel, 2013; Herbette et al., 2015). One can predict that the chemical nature of these substrata will affect disease outcome as the pathogen that do not have the adapted enzymatic tools to break down those polymers will likely display a decreased fitness and *in planta* colonization. For example, it has been shown that cell wall chemical composition directly affects the ability for vascular pathogens to colonize woody tree species (Blanchette, 1995). Comparative genomic studies suggested that pectinolytic enzymatic activity for breaking-down pectin-rich compounds such as tyloses walls and gels play a central role in the fitness of vascular wilts (Klosterman et al., 2011). Previous reports confirmed that *P. chlamydospora* could metabolize pectin while possessing a limited ability to break down cellulo-lignified secondary cell walls (Surico et al., 2001; Santos et al., 2006; Valtaud et al., 2009; Fischer et al., 2016). Computational analysis of the *P. chlamydospora* genome also supported these conclusions (Antonielli et al., 2014; Morales-Cruz et al., 2015). Our histological data concurred with those predictions showing that *P. chlamydospora* was able to progress actively in occluded vessels by colonizing pectin-rich gel pockets and outer tyloses walls.

Another important aspect of the host-pathogen interaction was the ability for *P. chlamydospora* to inhibit vessel occlusion. In some heavily colonized vessels, the compartmentalization process was initiated but failed to reach completion. Similar observations were also reported in other wilt pathosystems (Beckman and Roberts, 1995). A failed compartmentalization may stem from the pathogen ability to impair the defense cascade mechanism leading to tylosis formation. *P. chlamydospora* is known to produce virulence factors such as phytotoxins and peptidic effectors that accumulate locally in paratracheal parenchyma cells and are translocated systemically in the transpiration stream and inhibit host metabolism (Fleurat-Lessard et al., 2010; Luini et al., 2010; Pouzoulet et al., 2013a; Pierron et al., 2016). In addition, *P. chlamydospora* was shown to down regulate a transcript coding for ACC oxidase involved in the synthesis pathway of ethylene (Yacoub et al., 2016). Our data showed that failed xylem vessel occlusion was associated with additional local reactions within the surrounding parenchymatic tissues including cell wall deposition of suberin and non-structural phenolic compounds. Edification of suberized layers in ray parenchyma cells surrounding the infection was previously identified as an efficient barrier against *P. chlamydospora* lateral spread from a xylem fascicular portion to the next (Pouzoulet et al., 2013a, 2014). In addition we observed a depletion of starch in infected tissues that were dramatically decreased in infected non-occluded vessels. Starch is an energy reserve required for the activation of the host defense system and was often found to be depleted in diseased wood (Shigo and Tippett, 1981; Martin et al., 2005; Rolshausen et al., 2008). Rolshausen et al. (2008) also proposed that vascular pathogens could utilize starch for their own metabolic activities and *P. chlamydospora* is known to possess the enzymatic arsenal to metabolize starch (Mugnai et al., 1999; Santos et al., 2006). Interestingly, studies in DED showed that starch levels were higher in resistant cultivars suggesting that availability of starch reserves could affect the tree capacity for defensive responses (Martin et al., 2005).

Longevity and productivity of vineyards and orchards are key components to the grower’s economic success. Vascular wilt diseases such as grapevine esca have a significant negative economic impact by reducing vineyard lifespan, cumulative yield over the years, and fruit marketability while also increasing the management costs (Siebert, 2001; Kaplan et al., 2016). Intensive agriculture that uses high inputs (i.e., water, fertilizers) to yield fast and high economic returns can have long-term damaging effects. Xylem morphology during its developmental stage is responsive to environmental signals (Pouzoulet et al., 2014). Data showed that high irrigation and over fertilization regimes positively affected severity of DED, and esca disease in grapevine (Solla and Gil, 2002a; Marchi et al., 2006; Guerin-Dubrana et al., 2012; Jimenez-Diaz et al., 2012). The adoption of intensive practices also concurred with the apparition of issue associated to Verticillium wilt in olive (*O. europaea*) (Jimenez-Diaz et al., 2012). Planting resistant varieties and implementing cultural management guidelines that limit disease risks are key elements of a foundational program for sustainable agriculture.

## Author Contributions

JP designed the research, collected and analyzed the data and wrote the manuscript. ES and MS analyzed the data and complement the writing. PR conceived the original project and research plans and co-wrote the manuscript.

## Conflict of Interest Statement

The authors declare that the research was conducted in the absence of any commercial or financial relationships that could be construed as a potential conflict of interest.

## Footnotes

1 http://imagej.nih.gov
