# Supplementary material for: Xylem Vessel Diameter Affects the Compartmentalization of the Vascular Pathogen Phaeomoniella chlamydospora in Grapevine
Source: Front Plant Sci. 2017 Aug 21;8:1442. doi: 10.3389/fpls.2017.01442 (PMC5566965; doi:10.3389/fpls.2017.01442)
Supplement: Supplementary file 1 [file Table_1.DOCX]

Supplementary Table 1

**Xylem Vessel Diameter Affects the Compartmentalization of the Vascular Pathogen *Phaeomoniella chlamydospora* in Grapevine**

**Jérôme Pouzoulet^1^, Elia Scudiero^2^, Marco Schiavon^1^, Philippe E. Rolshausen^1^**

*** Correspondence:**

Philippe E. Rolshausen

Tel: +1 951 827 6988

Email: [philrols@ucr.edu](mailto:philrols@ucr.edu)

**Supplementary Table 1.** Foundation Plant Services (FPS, University of California, Davis) plant material used in this study**.**

**^(a)^**Number of canes analyzed per mother plants for the characterization of xylem morphology of *Vitis vinifera* cultivars.

| *V.vinifera* cv. | Year planted | FPS selection # | FPS accession | Year of experiment | Number of canes*^a^* |
| --- | --- | --- | --- | --- | --- |
| Thompson Seedless | 1995 | 02A | NYC C R2.00 26.00 | 2013 | 3 |
|  | 1995 | 02A | NYL C R2.00 27.00 | 2013 | 3 |
|  | 1995 | 02A | NYC C R2.00 16.00 | 2014 | 3 |
|  | 1995 | 02A | NYC C R2.00 17.00 | 2014 | 3 |
| Cabernet Sauvignon | 1998 | 31 | BKN D R3.00 5.00 | 2013 | 3 |
|  | 1998 | 31 | BKN D R3.00 9.00 | 2013 | 3 |
|  | 1998 | 31 | BKN D R3.00 5.00 | 2014 | 3 |
|  | 1998 | 31 | BKN D R3.00 9.00 | 2014 | 3 |
| Chardonnay | 1996 | 50 | NYL C R13.00 3.00 | 2013 | 3 |
|  | 1996 | 50 | NYL C R13.00 7.00 | 2013 | 3 |
|  | 1996 | 50 | NYL C R13.00 2.00 | 2014 | 3 |
|  | 1996 | 50 | NYL C R13.00 3.00 | 2014 | 3 |
| Merlot | 1996 | 6 | NYL C R8.00 15.00 | 2013 | 3 |
|  | 1996 | 6 | NYL C R8.00 17.00 | 2013 | 3 |
|  | 1996 | 6 | NYL C R8.00 12.00 | 2014 | 3 |
|  | 1996 | 6 | NYL C R8.00 13.00 | 2014 | 3 |
