# Supplementary material for: Xylem Vessel Diameter Affects the Compartmentalization of the Vascular Pathogen Phaeomoniella chlamydospora in Grapevine
Source: Front Plant Sci. 2017 Aug 21;8:1442. doi: 10.3389/fpls.2017.01442 (PMC5566965; doi:10.3389/fpls.2017.01442)
Supplement: Supplementary file 3 [file Table_3.DOCX]

Supplementary Table 3

**Xylem Vessel Diameter Affects the Compartmentalization of the Vascular Pathogen *Phaeomoniella chlamydospora* in Grapevine**

**Jérôme Pouzoulet^1^, Elia Scudiero^2^, Marco Schiavon^1^, Philippe E. Rolshausen^1^**

*** Correspondence:**

Philippe E. Rolshausen

Tel: +1 951 827 6988

Email: [philrols@ucr.edu](mailto:philrols@ucr.edu)

**Supplementary Table 3.** Multiple comparisons of standardized *Phaeomoniella chlamydospora* DNA in xylem fragments of four *Vitis vinifera* L. cvs distant from the inoculation point (L2; 15-30mm) for 2013 and 2014**.** Multiple comparisons were carried out using Fisher's protected least significant difference test at the 0.05 probability level. Multiple comparisons were selected according to the effects and interactions previously found between cultivar and year using ANOVA (n=64).

| **Cultivar** | **Year** | **Estimated *P.ch* DNA amount (fg/ng)** | **Statistical group** |
| --- | --- | --- | --- |
| Merlot | 2013 | 578 | D |
| Merlot | 2014 | 997 | D |
| Chardonnay | 2013 | 2755 | CD |
| Chardonnay | 2014 | 2272 | CD |
| Cabernet Sauvignon | 2013 | 6940 | B |
| Cabernet Sauvignon | 2014 | 2039 | CD |
| Thompson Seedless | 2013 | 12229 | A |
| Thompson Seedless | 2014 | 4750 | BC |
