# Supplementary material for: Xylem Vessel Diameter Affects the Compartmentalization of the Vascular Pathogen Phaeomoniella chlamydospora in Grapevine
Source: Front Plant Sci. 2017 Aug 21;8:1442. doi: 10.3389/fpls.2017.01442 (PMC5566965; doi:10.3389/fpls.2017.01442)
Supplement: Supplementary file 4 [file Table_4.DOCX]

Supplementary Table 4

**Xylem Vessel Diameter Affects the Compartmentalization of the Vascular Pathogen *Phaeomoniella chlamydospora* in Grapevine**

**Jérôme Pouzoulet^1^, Elia Scudiero^2^, Marco Schiavon^1^, Philippe E. Rolshausen^1^**

*** Correspondence:**

Philippe E. Rolshausen

Tel: +1 951 827 6988

Email: [philrols@ucr.edu](mailto:philrols@ucr.edu)

**Supplementary Table 4.** ANOVA outputs of the 20µm vessel diameter class in the dorso-ventral area of *Vitis vinifera* stems cvs. Merlot, Cabernet Sauvignon, Chardonnay and Thompson Seedless in 2013 and 2014 (n=48).

Multiple comparisons are displayed in Supplementary Table 3.

*Significant at the 0.05 probability level

**Significant at the 0.01 probability level

***Significant at the 0.001 probability level

| Effect | Vessel diameter classes (Pr > F) | | | | | | | |
| --- | --- | --- | --- | --- | --- | --- | --- | --- |
|  | 60-79 µm | 80-99 µm | 100-119 µm | 120-139 µm | 140-159 µm | 160-179 µm | 180-199 µm | >200 µm |
| Cultivar | 0.0522 | **0.0002***** | **0.001***** | **0.0891** | **<0.0001***** | **0.0002***** | **0.0186**** | **0.0403*** |
| Year | 0.9561 | 0.6543 | 0.6549 | 0.4249 | 0.2978 | 0.2537 | 0.1728 | **0.0362*** |
| Cultivar*Year | **0.0057**** | 0.0556 | 0.2308 | 0.8694 | 0.2205 | 0.2845 | 0.1043 | **0.0257*** |
