# Supplementary material for: Xylem Vessel Diameter Affects the Compartmentalization of the Vascular Pathogen Phaeomoniella chlamydospora in Grapevine
Source: Front Plant Sci. 2017 Aug 21;8:1442. doi: 10.3389/fpls.2017.01442 (PMC5566965; doi:10.3389/fpls.2017.01442)
Supplement: Supplementary file 5 [file Table_5.docx]

Supplementary Table 5

**Xylem Vessel Diameter Affects the Compartmentalization of the Vascular Pathogen *Phaeomoniella chlamydospora* in Grapevine**

**Jérôme Pouzoulet^1^, Elia Scudiero^2^, Marco Schiavon^1^, Philippe E. Rolshausen^1^**

*** Correspondence:**

Philippe E. Rolshausen

Tel: +1 951 827 6988

Email: [philrols@ucr.edu](mailto:philrols@ucr.edu)

**Supplementary Table 5.** Multiple comparisons of the 20µm vessel diameter class in the dorso-ventral area of *Vitis vinifera* stems cvs. Merlot, Cabernet Sauvignon, Chardonnay and Thompson Seedless in 2013 and 2014**.** Multiple comparisons were carried out using Fisher's protected least significant difference test at the 0.05 probability level. Multiple comparisons were selected according to the effects and interactions previously found between cultivar and year using ANOVA (n=48). M= Merlot; Ch= Chardonnay; CS= Cabernet Sauvignon; TS= Thomson Seedless.

| Class | Effect | Cultivar | Year | Estimated number of vessel / mm^2^ | Statistical group |
| --- | --- | --- | --- | --- | --- |
| 60-79 | Cultivar*Year | CS | 2013 | 3.7896 | C |
| 60-79 | Cultivar*Year | CS | 2014 | 2.8108 | C |
| 60-79 | Cultivar*Year | Ch | 2013 | 3.2819 | C |
| 60-79 | Cultivar*Year | Ch | 2014 | 4.0004 | BC |
| 60-79 | Cultivar*Year | M | 2013 | 5.9743 | A |
| 60-79 | Cultivar*Year | M | 2014 | 4.3328 | BC |
| 60-79 | Cultivar*Year | TS | 2013 | 3.7826 | C |
| 60-79 | Cultivar*Year | TS | 2014 | 5.61 | AB |
| 80-99 | Cultivar | CS | _ | 3.798 | C |
| 80-99 | Cultivar | Ch | _ | 6.4102 | AB |
| 80-99 | Cultivar | M | _ | 7.6395 | A |
| 80-99 | Cultivar | TS | _ | 5.4195 | B |
| 100-119 | Cultivar | CS | _ | 4.7534 | B |
| 100-119 | Cultivar | Ch | _ | 5.2365 | B |
| 100-119 | Cultivar | M | _ | 8.9174 | A |
| 100-119 | Cultivar | TS | _ | 5.5513 | B |
| 140-159 | Cultivar | CS | _ | 1.8378 | B |
| 140-159 | Cultivar | Ch | _ | 1.6076 | BC |
| 140-159 | Cultivar | M | _ | 0.8254 | C |
| 140-159 | Cultivar | TS | _ | 3.6166 | A |
| 160-179 | Cultivar | CS | _ | 1.2275 | A |
| 160-179 | Cultivar | Ch | _ | 0.4787 | B |
| 160-179 | Cultivar | M | _ | 0.02125 | B |
| 160-179 | Cultivar | TS | _ | 1.5873 | A |
| 180-199 | Cultivar | CS | _ | 0.4985 | AB |
| 180-199 | Cultivar | Ch | _ | 0.09588 | BC |
| 180-199 | Cultivar | M | _ | <0.0001 | C |
| 180-199 | Cultivar | TS | _ | 0.7325 | A |
| >200 | Cultivar*Year | CS | 2013 | 0.269 | B |
| >200 | Cultivar*Year | CS | 2014 | 0.09532 | B |
| >200 | Cultivar*Year | Ch | 2013 | <0.0001 | B |
| >200 | Cultivar*Year | Ch | 2014 | <0.0001 | B |
| >200 | Cultivar*Year | M | 2013 | <0.0001 | B |
| >200 | Cultivar*Year | M | 2014 | <0.0001 | B |
| >200 | Cultivar*Year | TS | 2013 | 1.607 | A |
| >200 | Cultivar*Year | TS | 2014 | 0.05289 | B |
